# Education and continued professional development

**DOI:** 10.1007/s00068-025-02831-9

**Published:** 2025-04-07

**Authors:** Christina Gaarder, Pål Aksel Naess, Ingo Marzi, Falco Hietbrink

**Affiliations:** 1 Department of Traumatology, Oslo University Hospital, Oslo, Norway; 2 Department of Trauma Surgery, University Medical Center Utrecht, Utrecht, The Netherlands; 3 Department of Trauma Surgery and Orthopedics, University Hospital, Goethe University Frankfurt, Frankfurt am Main, Germany

## Abstract

Trauma care requires a multidisciplinary approach, with surgeons ensuring timely and effective treatment for severely injured patients while collaborating closely with intensivists, emergency physicians, and rehabilitation teams. In addition to advanced surgical skills, trauma surgeons develop non-technical competencies such as leadership, communication, and decision-making to coordinate care effectively. This chapter addresses the challenges of maintaining trauma surgical competence in Europe, focusing on essential training programmes, quality improvement initiatives, and interdisciplinary collaboration. It also examines the impact of an ageing population, the integration of new technologies, and the vital role of surgical involvement in intensive care units (ICUs). Structured education and continuous professional development are critical to improving outcomes for trauma patients. ESTES, Polytrauma, Whitebook.

## Introduction

Trauma care is a complex field requiring surgeons to apply advanced skills, critical decision-making, and effective teamwork to manage severely injured patients. Surgeons guide care from initial stabilisation to rehabilitation, making their expertise essential for achieving positive outcomes. Across Europe, maintaining trauma surgical competence poses challenges due to varying healthcare systems and training frameworks.

This chapter, based on *What Trauma Patients Need: The European Dilemma*, outlines the core educational and professional requirements for trauma surgeons. It details the technical and non-technical skills necessary for managing trauma patients, emphasises the importance of multidisciplinary collaboration, and highlights the role of quality improvement processes. Additionally, the chapter explores challenges such as an ageing population and the adoption of new technologies, underscoring the need for adaptable and comprehensive training to meet the evolving demands of trauma care.

### Surgical knowledge and skills

The complex and acute nature of trauma care demands a broad understanding of physiology, anatomy, and surgical techniques, combined with non-technical skills such as communication, leadership, and organisational expertise. Multidisciplinary and interdisciplinary collaboration is essential for improving outcomes across the trauma care continuum.

### Non‑technical skills: leadership and teamwork

Leadership, communication, and logistical management are fundamental for trauma surgeons. Trauma care inherently requires a team-based, multidisciplinary approach. In high-pressure situations, pre-established routines and a standardised mindset for surgical decision-making are essential.

Regular simulated team practice should be a cornerstone of training to reinforce these routines. As Archilochus aptly stated, ‘We don’t rise to the level of our expectations; we fall to the level of our training.’ Training and standardisation must extend beyond the emergency department, encompassing all phases of care, from admission to rehabilitation.

Trauma surgeons guide the patient through the entire care pathway, ensuring coordination among various teams. Leadership is particularly vital during transitions of care and in the emergency department, where early decisions have long-lasting consequences.

### Timing and decision-making

Trauma care requires precise, timely decisions tailored to the patient’s physiology and injury profile. Key considerations include:

1. Assessment of Interventions:

- Determine whether surgical or non-surgical management is appropriate based on the patient’s condition and injury severity.

2. Prioritisation and Timing:

- Balance the urgency of interventions with the patient’s physiological tolerance to ensure optimal outcomes.
- Consider a stepwise approach to procedures when necessary.

3. Extent of Surgical Procedures:

- Tailor the scope of surgery to minimise the physiological burden on the patient.
- Recognise when damage control surgery is required as an initial stabilisation measure before definitive management.

4. Decision Not to Operate:

- Understand that the decision to delay or omit surgery can be equally critical and should be based on the patient’s overall condition, including immunological and physiological factors.

5. Surgeon’s Expertise:

- Develop proficiency in understanding and managing the physiological impacts of interventions.
- Apply specialised knowledge of resuscitation, critical care support, and trauma-specific surgical techniques.

Effective trauma care requires surgeons to balance urgency with precision, tailoring interventions to the patient’s unique physiology and injury profile. Mastery of timing, decision-making, and damage control surgery, along with understanding the physiological impact of procedures, begins in residency and is refined through fellowships and ongoing professional development. The goal remains achieving confidence to make lifesaving decisions in the moment.

### Training programmes

Core competencies for trauma surgeons can be developed through internationally recognised courses, including:

- **ATLS/ETC**: Foundational skills for managing severely injured patients.
- **DSTC/DSATC**: Advanced trauma surgery techniques, focusing on critical decision-making and technical expertise.
- **ATOM and ASSET**: Essential surgical skills for complex injuries.

These courses establish a minimum skill set for trauma care. Additional training tailored to regional needs, such as lifesaving neurosurgical procedures or vascular shunting, may be necessary. Trauma surgeons should also be proficient in stabilising extremity injuries and managing vascular trauma during resuscitation.

### Quality improvement programmes

Continuous quality improvement is essential for optimising trauma care. Regular analysis of patient pathways and feedback on team performance ensure effective learning and adaptation. Key practices include:

- Mortality and morbidity meetings.
- Trauma-specific debriefings involving all team members.
- Identification of improvement areas, such as communication, updated protocols, and technical skills training.
- Regular trauma team simulations to reinforce crew resource management (CRM).

A structured approach can be summarized with the following formula:

$$\eqalign{{\rm{Competence = }} & {\rm{Modular}}\,{\rm{ training }}\,{\rm{and}} \cr & {\rm{basic}}\,{\rm{skill}}\,{\rm{ maintenance}} \cr & {\rm{ \times Patient}}\,{\rm{ journey}}\,{\rm{ analysis }} \cr & {\rm{ \times Ongoing}}\,{\rm{ knowledge}}\,{\rm{ updates}} \cr} $$

This formula underscores the interconnected elements required for trauma surgeons to develop and maintain expertise. For instance, modular training in decision-making can be reinforced through regular pathway reviews, ensuring surgeons are equipped to address both common and emerging challenges.

These processes ensure all stakeholders focus on the injured patient as a unique entity while enabling discussions and implementation of new strategies. Quality improvement is an ongoing process, integrating feedback to refine protocols and improve outcomes.

### Challenges of an ageing population

The ageing population across Europe introduces new challenges for trauma care, including:

- Fragile bones and comorbidities.
- Increased use of anticoagulants and associated risks.
- Atherosclerotic vascular disease complicating recovery.

Simultaneously, the rapid introduction of new techniques and devices necessitates careful evaluation. Trauma surgeons must critically assess the risks, indications, and timing of these innovations, balancing patient safety with cost-effectiveness. Continuing education ensures surgeons remain equipped to address these challenges.

### Trauma patients in the ICU

ICUs in Europe are predominantly staffed by intensivists with backgrounds in anaesthesiology, internal medicine, neurology, or cardiology. Surgeons offer complementary expertise in surgical decision-making and trauma-specific physiology.

Effective surgical contributions to trauma care in the ICU require combined generalist medical knowledge and specific expertise in trauma care. Unfortunately, training in intensive care medicine within surgical residency programmes across Europe is often limited, and insufficient for mastering the complexities of managing critically ill trauma patients.

### Addressing gaps in surgical ICU training

To improve surgical contributions in the ICU:

- Residency programmes should integrate intensive care training, emphasising the physiological principles underlying trauma management.
- Fellowships or scholarships should provide advanced critical care training for trauma surgeons.
- Flexible pathways are needed to address time and workload constraints imposed by operative practice and European working time regulations.

### ICU formats and collaboration

Closed-format ICUs, led by intensivists, have demonstrated reduced morbidity and mortality. This approach benefits from integrated care, avoiding segmentation by organ systems, and is applicable to trauma patients, who require coordinated, multidisciplinary care throughout their recovery.

Regardless of the ICU format—closed, open, or mixed—surgical involvement remains critical. Collaboration between surgeons and intensivists ensures trauma patients receive coordinated, multidisciplinary care tailored to their needs.

### Conclusion and needs for the future

While trauma systems and training structures differ across Europe, the need for consistent, high-quality care for severely injured patients remains universal. Effective trauma management relies on multidisciplinary teams, with surgeons central to leadership, decision-making, and patient care.

To address evolving challenges such as an ageing population and advancing technology, surgical education must integrate technical skills, physiology, communication, and teamwork. Comprehensive training programmes and fellowships should be paired with ongoing quality improvement initiatives and interdisciplinary collaboration, particularly in intensive care settings.

As trauma care evolves, surgical expertise in both operative and non-operative management must be ensured. Trauma surgeons must be recognised as specialists in critical care decision-making and strengthen partnerships with intensivists to deliver integrated, patient-centred care. Future efforts should prioritise training, collaboration, and quality improvement to meet the demands of modern trauma care across Europe.

## Data Availability

No datasets were generated or analysed during the current study.
